# Clinical considerations on the use of evinacumab for the treatment of homozygous familial hypercholesterolemia

**DOI:** 10.1016/j.ajpc.2026.101790

**Published:** 2026-08-11

**Authors:** Archna Bajaj, Michael McGuire, Suzanne Shugg, Richard Godin, James A. Underberg

**Affiliations:** a Division of Translational Medicine and Human Genetics, University of Pennsylvania, Philadelphia, PA, USA; b Regeneron Pharmaceuticals, Inc., Tarrytown, NY, USA; c NJ Cardiology Associates, West Orange, NJ, USA; d NYU Langone Health, New York, NY, USA

**Keywords:** Homozygous familial hypercholesterolemia, Evinacumab, ANGPTL3, Atherosclerotic cardiovascular disease

## Abstract

Homozygous familial hypercholesterolemia (HoFH) is a rare genetic disorder that results in severely elevated low-density lipoprotein cholesterol (LDL-C) due to dysfunctional activity of the low-density lipoprotein receptor (LDLR) and impaired clearance of LDL-C. Individuals with HoFH require intensive lipid-lowering therapies (LLTs), ideally starting in early childhood, to aggressively lower LDL-C levels and mitigate risk of early-onset atherosclerotic cardiovascular disease caused by lifetime exposure to high LDL-C levels. However, most conventional LLTs lower LDL-C through an LDLR-dependent mechanism, limiting their effectiveness in individuals with HoFH, who have little to no LDLR activity. Evinacumab is a fully human monoclonal antibody against angiopoietin-like protein 3 (ANGPTL3), and has an LDLR-independent mechanism of action. By inhibiting ANGPTL3, evinacumab increases the activity of lipoprotein lipase and endothelial lipase, consequently increasing the clearance of LDL precursors and lowering LDL-C levels. Following its initial US Food and Drug Administration approval in 2021, evinacumab has been approved to treat individuals with HoFH from 1 year of age in the US and as young as 6 months of age in multiple other countries. Here, we describe the role of evinacumab in the HoFH treatment landscape, summarize the available clinical evidence for evinacumab, and provide practical guidance on its use from the clinician’s perspective.

## 1 Introduction

Evinacumab is a fully human monoclonal antibody that targets angiopoietin-like protein 3 (ANGPTL3). It is approved in multiple countries for adults, adolescents, and children with homozygous familial hypercholesterolemia (HoFH) [[1], [2], [3], [4], [5]]. HoFH is a rare disorder characterized by extreme elevations of low-density lipoprotein cholesterol (LDL-C) caused by impaired activity of the low-density lipoprotein receptor (LDLR), which then impairs binding and removal of LDL-C from circulation [6]. Most standard-of-care lipid-lowering therapies (LLTs) utilize the LDLR pathway, limiting their effectiveness in people with HoFH who often have little to no functional LDLR activity [7]. By targeting ANGPTL3, an inhibitor of lipoprotein lipase (LPL) and endothelial lipase (EL), evinacumab increases activity of these lipases, which in turn enhances clearance of precursors of LDL, such as very low-density lipoprotein (VLDL) [8]. The mechanism of action of evinacumab bypasses the LDLR pathway, allowing it to be effective independently of LDLR activity [6,8]. Here, we describe the role of evinacumab in the HoFH treatment landscape, summarize the available clinical evidence for evinacumab, and provide practical guidance on its use from the clinician’s perspective.

## 2 Introduction to HoFH

### 2.1 HoFH definition and diagnosis

HoFH is a rare disorder with an estimated prevalence of ∼1:160,000–300,000, characterized by extreme elevations of LDL-C from birth [9,10]. Individuals with HoFH are at high risk for developing premature atherosclerotic cardiovascular disease (ASCVD), which can frequently result in early mortality [11].

HoFH can be diagnosed clinically by the detection of severely elevated LDL-C levels with untreated levels frequently exceeding 400 mg/dL, in conjunction with manifestations of hypercholesterolemia such as xanthomas, corneal arcus, or premature ASCVD, or a family history consistent with familial hypercholesterolemia (FH) or premature ASCVD [12] (Box 1). Furthermore, individuals with HoFH may not respond to standard-of-care LLTs and LDL-C levels may remain elevated despite multiple LDL-C–lowering therapies [12]. In addition to a clinical diagnosis, HoFH can also be diagnosed through detection of identifiable genetic variants associated with loss of LDLR function, including in the *LDLR, APOB, PCSK9*, or *LDLRAP1* genes [12]. However, while many genetic variants implicated in HoFH have been identified, diagnosis via genetic testing is limited by the fact that there may be as yet unidentified genetic variants that are causal for the disease phenotype [9,12]. Therefore, while a positive genetic test provides confirmation of the HoFH diagnosis, a negative genetic test does not exclude a diagnosis, and contemporary guidelines state that genetic testing is not required for diagnosis or clinical management of HoFH [9,13]. As our understanding of the genetic complexity of this disease has evolved, so has the terminology used to describe FH. Current guidelines now recognize not only true homozygotes, but also compound heterozygotes and double heterozygotes, all of which can produce a severe FH phenotype consistent with HoFH [9,12].

### 2.2 Current treatments for people with HoFH

While the presence of other risk factors may impact the development of ASCVD in HoFH, risk is primarily driven by exposure to severely elevated LDL-C starting at birth [9]. Early treatment and sustained lifelong management are therefore necessary to mitigate the risk of ASCVD and its related morbidity and mortality [9,12,14,15]. While maintaining a healthy lifestyle (i.e., diet and physical activity) is recommended, most people with HoFH will require aggressive pharmacologic interventions with multiple LLTs and sometimes non-pharmacologic intervention with lipoprotein apheresis to reach acceptable levels of LDL-C (Table 1) [9,12,[14], [15], [16], [17], [18], [19], [20], [21], [22], [23], [24], [25], [26], [27], [28]]. Early, effective, and lifelong treatment could restore near-normal life expectancy in people with HoFH [29]. Various national and international cardiology societies provide guidance for the treatment of HoFH, including recent guidance from the European Society of Cardiology (ESC) in 2023 and the ESC and European Atherosclerosis Society (EAS) in 2025, the consensus from the National Lipid Association (NLA) in 2026, and the clinical practice guidelines from the American College of Cardiology (ACC) and American Heart Association (AHA) Joint Committee in 2026 (Fig. 1) [9,12,15,[30], [31], [32], [33], [34]]. These guidelines recommend rapid intensification of pharmacotherapy for individuals with HoFH, with LDL-C goals of <70 mg/dL for those without ASCVD, <55 mg/dL for those with ASCVD, and <40 mg/dL for those with ASCVD and recurrent vascular events; it has been observed that the risk of ASCVD events is proportional to the achieved LDL-C concentration down to <20 mg/dL, without additional safety concerns [9,12,34].

**Table 1:** Approved LDL-C lowering therapies and efficacy in people with HoFH.

Agent	Mechanism of action	Effects on LDL-C in HoFH, %	LDLR-independent	Guideline recommendation (NLA 2026 consensus statement) [9]
Statins [14,16]	HMG-CoA reductase inhibition	15–26	No	First-line treatment in combination with ezetimibe
Ezetimibe [17]	NPC1L1 inhibition	19	No	First-line treatment in combination with statins
PCSK9 inhibitors [[18], [19], [20]]	PCSK9 inhibition	Evolocumab 420 mg Q2W or Q4W: 21–31 Alirocumab 150 mg Q2W: 27	No	Add-on to statin + ezetimibe^b^
Lomitapide [21]	MTP inhibition	50	Yes	Adjunct to standard LLT in people aged ≥18 years^c^
Evinacumab^a^	ANGPTL3 inhibition	40–50	Yes	Adjunct to standard LLT in adults and children from 1 year and older in the US and from 6 months of age and older in multiple other countries

^a^See Table 2 for full list of supporting references.

^b^If there is no response to treatment after 1–2 doses (≤15%), consider discontinuing PCSK9 inhibitor, and initiate other LLT.

^c^FDA approved for use in children 2 years and older as of March 3, 2026.

ANGPTL3, angiopoietin-like protein 3; EAS, European Atherosclerosis Society; FDA, Food and Drug Administration; HMG-CoA, β-Hydroxy β-methylglutaryl-CoA β-Hydroxy β-methylglutaryl-CoA; LDL-C, low density lipoprotein cholesterol; LDLR, low density lipoprotein receptor; LLT, lipid-lowering therapy; MTP, microsomal triglyceride transfer protein; NPC1L1, Niemann-Pick C1-like 1 protein; PCSK9, proprotein convertase subtilisin/kexin 9; Q2W, every 2 weeks; Q4W, every 4 weeks.

**Fig. 1:**
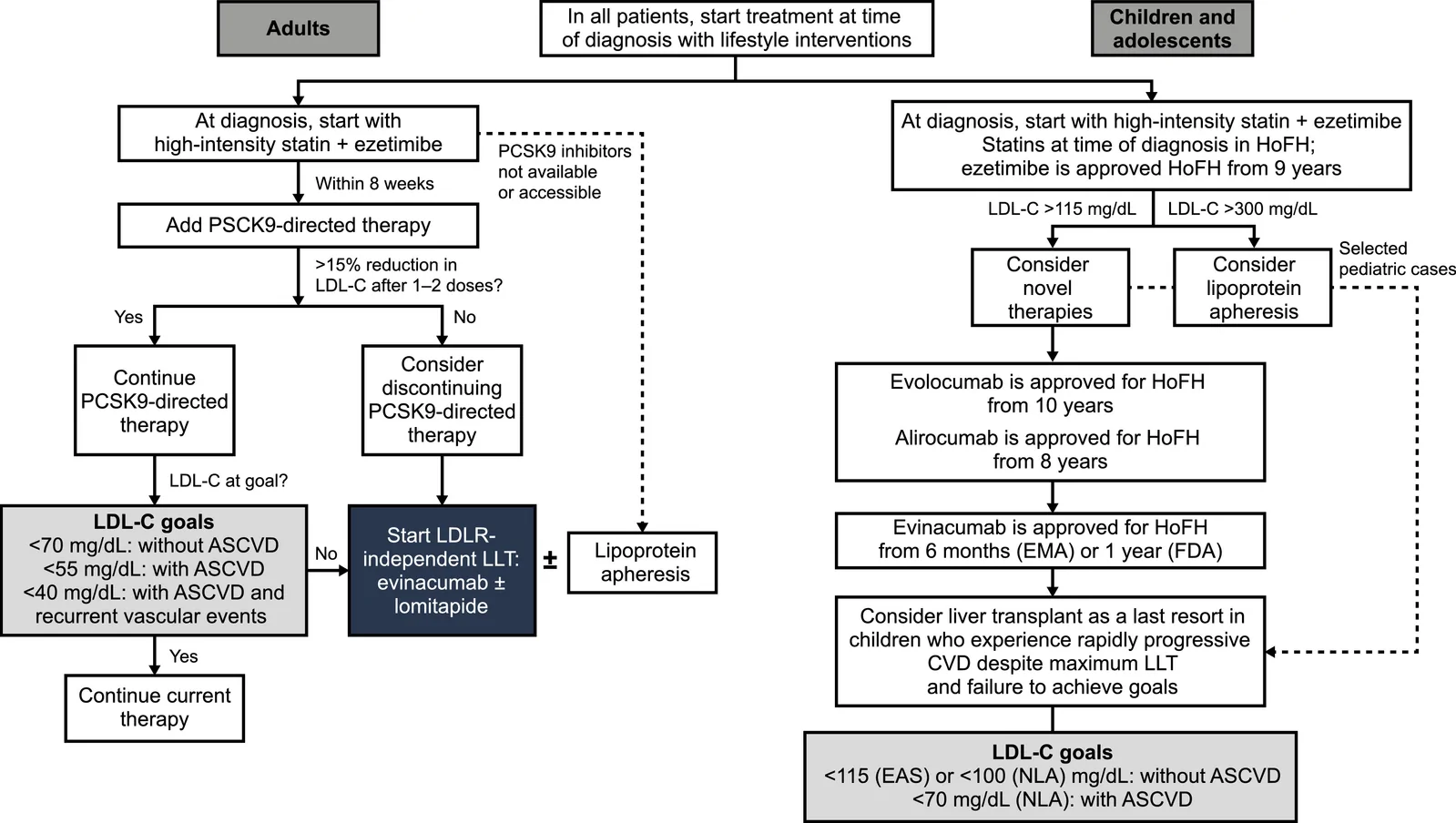
Treatment guidelines for HoFH based on the 2026 expert clinical consensus from the National Lipid Association, the 2023 consensus statement from the European Atherosclerosis Society, and the 2026 American College of Cardiology/American Heart Association Joint Committee clinical practice guidelines on the management of dyslipidemia [9,12,15]. ASCVD, atherosclerotic cardiovascular disease; CVD, cardiovascular disease; EMA, European Medicines Agency; FDA, US Food and Drug Administration; HoFH, homozygous familial hypercholesterolemia; LDL-C, low-density lipoprotein cholesterol; LDLR, low-density lipoprotein receptor; LLT, lipid-lowering therapy; PCSK9, proprotein convertase subtilisin/kexin type 9.

Statin treatment with the addition of ezetimibe is the first line of therapy for people with HoFH, followed by the addition of proprotein convertase subtilisin/kexin type 9 (PCSK9) inhibitors and other non-statin therapies [9,12]. However, these therapies are LDLR-dependent, and as HoFH is caused by severely impaired LDLR function, they are unlikely to yield satisfactory LDL-C reduction [20]. Thus, many people with HoFH will require additional LLTs beyond these conventional LDLR-dependent therapies to achieve their LDL-C goals [6,7,12,15].

Lipoprotein apheresis is a non-pharmacologic and LDLR-independent intervention that effectively removes LDL-C from circulation and may be considered for individuals with HoFH. However, lipoprotein apheresis must be done at regular intervals (typically weekly) as there is a rapid return to baseline LDL-C levels in the absence of repeated treatment [12,35,36]. Additionally, apheresis is an invasive procedure that is only available in specialized treatment centers, which are scarce in many countries [12,36,37]. In terms of pharmacologic approaches to HoFH treatment that do not depend on the LDLR pathway, 2 therapies with differing mechanisms of action are currently approved: lomitapide, an inhibitor of microsomal triglyceride-transfer protein; and evinacumab, a monoclonal antibody targeting ANGPTL3. Evinacumab is recommended as an LDLR-independent therapy in adults and children with HoFH [15] and is the subject of this review.

## 3 Clinical development of evinacumab

### 3.1 ANGPTL3 as a therapeutic target for HoFH

ANGPTL3 is a member of the angiopoietin-like protein family that is expressed mainly in the liver and plays a role in the regulation of lipid metabolism as an inhibitor of LPL and EL, enzymes involved in the hydrolysis of the triglyceride and phospholipid content of VLDLs, respectively. Inhibiting the activity of these lipases results in increased production of LDL particles [38]. ANGPTL3 gained interest as a therapeutic target for HoFH after it was discovered that deficiency in ANGPTL3, which is a cause of familial combined hypolipidemia, is associated with a substantial reduction in lipoprotein levels (including LDL-C) and cardiovascular events [[39], [40], [41], [42], [43]]. In a study of 45,226 individuals who provided genetic data as part of a large European study (DiscovEHR), differences in serum lipid measures between people with and without *ANGPTL3* loss-of-function variants were examined. Those who were heterozygous for loss-of-function variants had 9% lower LDL-C levels and a 41% lower risk of coronary artery disease than those without such variants [43]. In another study of 20,092 people with available plasma lipid levels from the Myocardial Infarction Genetics Consortium, a large, international research collaboration that pools data from genetic studies worldwide to find genetic risk factors for myocardial infarction and coronary artery disease, carriers of *ANGPTL3* loss-of-function variants had 12% lower LDL-C levels than those without these variants, alongside a 34% reduced risk of coronary artery disease. This study also matched 3 individuals with complete ANGPTL3 deficiency to 3 first-degree relatives without *ANGPTL3* variants and observed very low levels of plasma lipid concentrations across a 20-year period in the 3 individuals with complete ANGPTL3 deficiency compared with matched relatives [39]. In another family-based study of 2 relatives with familial combined hypolipidemia, loss-of-function *ANGPTL3* variants were observed in each individual, highlighting the role of this gene in LDL-C metabolism [42].

Taken together, these studies highlighted the apparent role of ANGPTL3 in lipoprotein clearance and supported the development of therapies that inhibit ANGPTL3 to reduce LDL-C levels and the associated risk for cardiovascular events.

### 3.2 Evinacumab as an LDLR-independent treatment for people with HoFH

Early preclinical studies demonstrating reduced production of LDL precursors through ANGPTL3 inhibition (Fig. 2) provided further evidence to enable evinacumab to progress to clinical investigation for HoFH [8]. Subsequent studies in healthy volunteers and people with hypertriglyceridemia (including severe hypertriglyceridemia) and heterozygous FH reported reductions in LDL-C, high-density lipoprotein cholesterol (HDL-C), total cholesterol, VLDL cholesterol, non-HDL-C, apolipoproteins A, B and C-III, triglycerides, and lipoprotein(a) with evinacumab treatment [44]. Clinical studies in healthy volunteers and in adult, adolescent, and pediatric participants with HoFH followed, as well as real-world studies carried out since regulatory approval, all of which have demonstrated the efficacy of evinacumab in reducing LDL-C alongside a well-tolerated safety profile [22–28,43,45] (Fig. 3; Table 2).

**Fig. 2:**
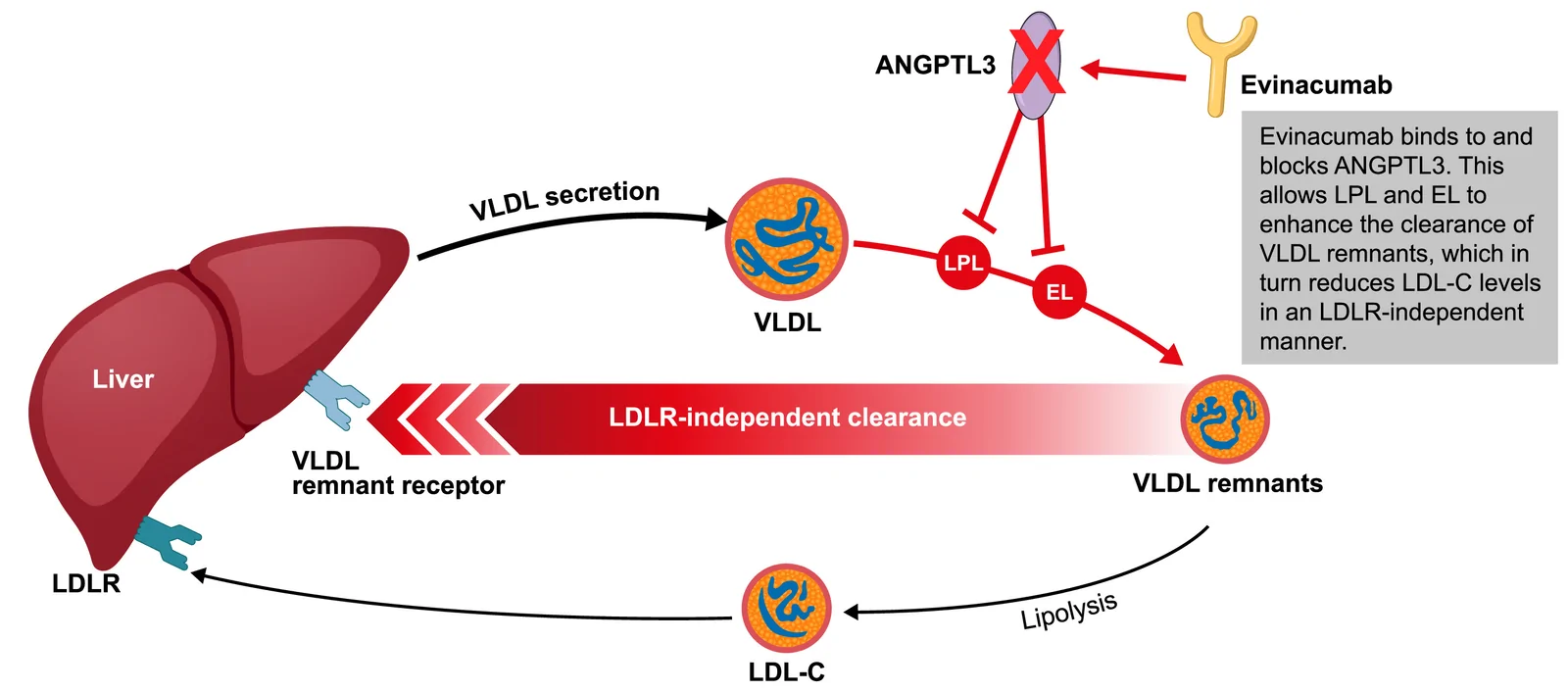
Mechanism of action for evinacumab. Evinacumab reduces LDL-C through ANGPTL3 inhibition. Specifically, ANGPTL3 inhibits 2 vascular lipases, LPL and EL, which are crucial for the catabolism of LDL-C and its precursors, very low-density lipoproteins [40,43,46]. By blocking ANGPTL3, evinacumab activates LPL and EL and consequently reduces the amount of LDL-C and its precursors in circulation, thereby lowering overall LDL-C levels independent of LDLR function [8]. ANGPTL3, angiopoietin-like protein 3; EL, endothelial lipase; IDL, intermediate-density lipoprotein; IDLR, intermediate-density lipoprotein receptor; LDL, low-density lipoprotein; LDL-C, low-density lipoprotein cholesterol; LDLR, low-density lipoprotein receptor; LPL, lipoprotein lipase; VLDL, very low-density lipoprotein.

**Fig. 3:**
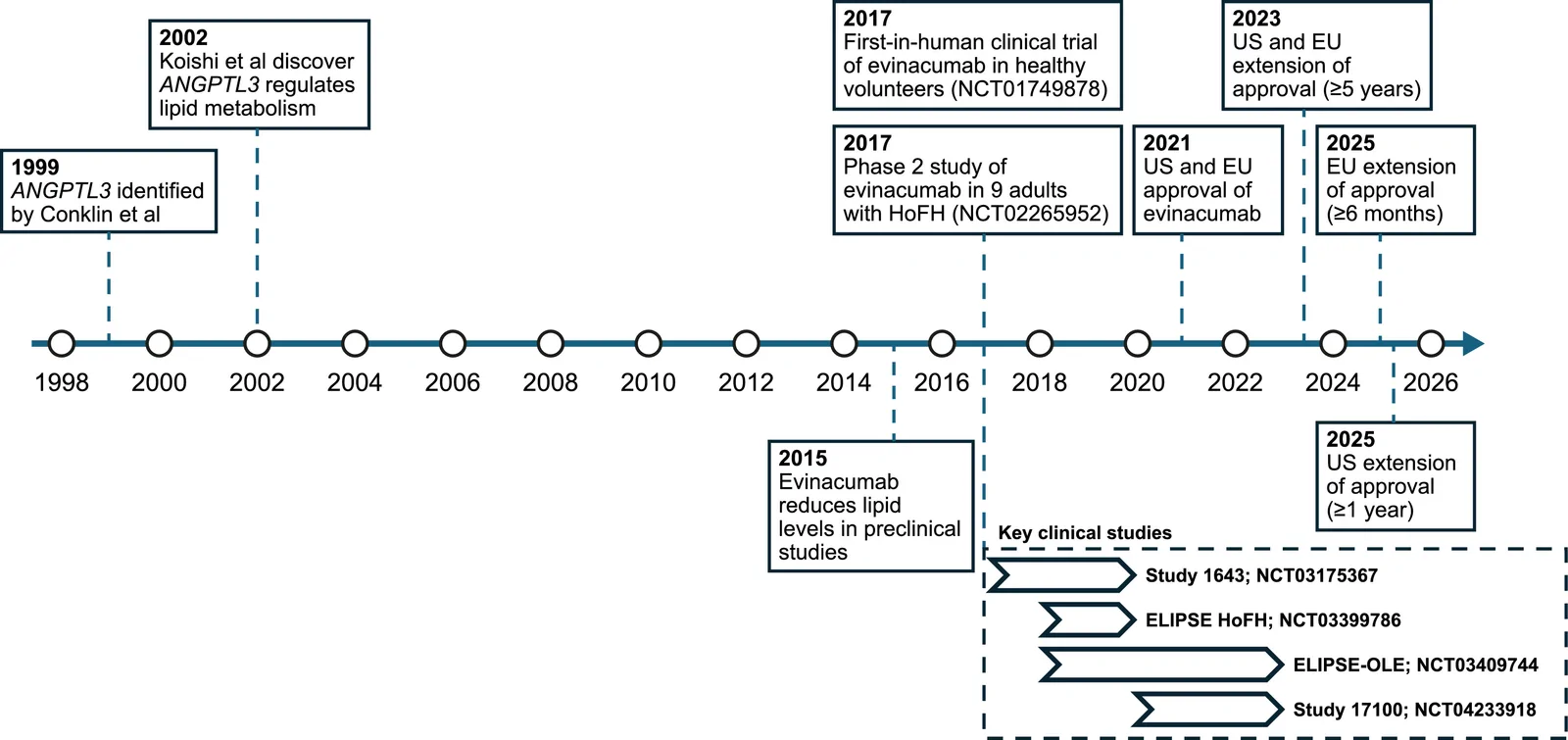
Development history of evinacumab [8,[22], [23], [24], [25], [26], [27], [28],^43^,^47^,^48^]. HoFH, homozygous familial hypercholesterolemia; OLE, open-label extension.

**Table 2:** Key clinical trial data for evinacumab.

Study name (trial registration)	Study design and participants	Evinacumab dose	Mean LDL-C change, %	Safety
Study of REGN1500 in participants with HoFH (NCT02265952) [22]	Phase 2, open-label, proof-of-concept study *n* = 9 participants aged ≥18 years with HoFH	75 mg SC; 150 mg SC; 250 mg SC; 5 mg/kg IV; 10 mg/kg IV; 20 mg/kg IV	−49	100% experienced ≥1 TEAE; no TEAEs led to treatment discontinuation
Study of evinacumab in participants with persistent hypercholesterolemia (Study 1643; NCT03175367) [23]	Phase 2, double-blind, placebo-controlled study *n* = 272 participants aged ≥18 years with refractory hypercholesterolemia (*n* = 75 placebo; *n* = 197 evinacumab)	450 mg weekly SC (*n* = 40); 300 mg weekly SC (*n* = 43); 300 mg biweekly SC (*n* = 39); placebo SC (*n* = 41); 15 mg/kg Q4W IV (*n* = 39); 5 mg/kg Q4W IV (*n* = 36); placebo IV (*n* = 34)	−50.5 (15 mg/kg vs. placebo cohorts; *P* < 0.001)	84% in the 15 mg/kg IV cohort experienced ≥1 TEAE; 16% experienced ≥1 serious TEAE; 2 TEAEs led to treatment discontinuation
ELIPSE HoFH (NCT03399786) [24,25]	Phase 3, double-blind, placebo-controlled study *n* = 65 participants aged ≥12 years with HoFH (*n* = 22 placebo; *n* = 43 evinacumab)	15 mg/kg IV Q4W	−49.0 (vs. placebo; *P* < 0.001)	TEAEs were similar between treatment cohorts; influenza-like illness was reported in 11% of participants in the evinacumab arm versus 0 in the placebo arm
ELIPSE-OLE (NCT03409744) [26]	Phase 3, single-arm, open-label extension study *n* = 116 participants aged ≥12 years with HoFH	15 mg/kg IV Q4W	−43.6	80.2% experienced ≥1 TEAE; 8.6% experienced treatment-related TEAEs
17,100 (NCT04233918) [27,28]	Phase 3, 3-part, open-label study Part B: *n* = 14 participants aged 5–11 years with HoFH Part C: *n* = 20 participants aged 5–11 years with HoFH	15 mg/kg IV Q4W	−48.3 (Part B) −40.7 (Part C)	Part B: 71.4% experienced ≥1 TEAE; 14.3% experienced treatment-related TEAEs Part C: 100% experienced ≥1 TEAE; no serious treatment-related TEAEs occurred

HoFH, homozygous familial hypercholesterolemia; IV, intravenous; LDL-C, low-density lipoprotein cholesterol; Q4W, every 4 weeks; SC, subcutaneous; TEAE, treatment-emergent adverse event.

### 3.3 Clinical trial data: efficacy

The first study of evinacumab in people with HoFH was a single-group, open-label phase 2 study involving 9 adults (NCT02265952) [22]. Participants were aged 18–47 years, 4 were female, and all were receiving LLTs at baseline; their on-treatment LDL-C at baseline ranged from 117 to 746 mg/dL. Evinacumab was administered at 250 mg subcutaneous (SC) for 1 dose at Week 0, 15 mg/kg intravenous (IV) for 1 dose at Week 2, and 450 mg SC weekly for 4 doses (Weeks 12–15). After 4 weeks of treatment, participants demonstrated a mean LDL-C of 157±90 mg/dL, a reduction of 49% [22].

Evinacumab treatment at a dose of 15 mg/kg IV every 4 weeks (Q4W) was then examined in 2 phase 3 studies enrolling adult (≥18 years) and adolescent (aged 12–<18 years) participants with HoFH: a double-blind, placebo-controlled study with a 24-week double-blind treatment period and a 24-week open-label treatment period (ELIPSE; NCT03399786); and a long-term open-label study (ELIPSE-OLE; NCT03409744) [24–26]. In ELIPSE, 63 adult and 2 adolescent participants were randomized 2:1 to evinacumab (*n* = 43) or placebo (*n* = 22) for 24 weeks, followed by a 24-week open-label treatment period in which participants assigned to placebo were allowed to cross over to receive evinacumab. In total, 54% were female, 74% were White, and 68% had a genetic diagnosis of HoFH. Despite background LLT, the mean LDL-C level at baseline was 255.1 mg/dL. Evinacumab treatment resulted in a between-group mean difference in LDL-C from baseline to Week 24 of 49.0% compared with placebo (*P* < 0.001) [24]. LDL-C reductions were maintained with long-term follow-up in those who received evinacumab (*n* = 64; mean decrease in LDL-C from baseline to Week 48 of 46.3%) [25]. ELIPSE-OLE included 116 participants (102 adults and 14 adolescents), 51% of whom were male, 69% were White, and 73% were genetically diagnosed. The mean LDL-C level at baseline was 261 mg/dL. The reduction in mean LDL-C from baseline to Week 24 was 43.6%, with reductions observed in both adult and adolescent participants (41.7% and 55.4%, respectively) [26].

An open-label study of evinacumab in pediatric participants aged 5–11 years with HoFH was also conducted (study 17,100; NCT04233918) [27,28]. During Part A (a phase 1b, single-dose, open-label study to assess safety, pharmacokinetics, and pharmacodynamics), 6 participants received a single 15 mg/kg IV dose of evinacumab. In Part B (a phase 3, single-arm, open-label study assessing efficacy, safety, and pharmacokinetics), 14 participants with a median age of 9.5 years and a mean LDL-C of 263.7 mg/dL at baseline were treated at a dose of 15 mg/kg IV Q4W. Participants had a reduction in LDL-C from baseline to Week 24 of 48.3% [27]. In Part C (a 48-week open-label extension study with an optional 24-week follow-up period), 20 participants from Parts A and B received evinacumab treatment for at least 48 weeks at a dose of 15 mg/kg IV Q4W. The mean LDL-C at baseline was 301.9 mg/dL and reductions from baseline to Weeks 48 and 72 were 44.6% and 40.7%, respectively. Evinacumab was generally well tolerated [28]. Treatment-emergent adverse events (TEAEs) occurred in all 20 participants. Two serious TEAEs were reported in 2 participants, neither of which were related to study drug. No treatment discontinuations or deaths were reported.

The use of evinacumab in children and adolescents has also been studied in Study 17,100 and the open-label extension (OLE) of ELIPSE-OLE. Participants aged 5–11 years (Study 17,100) and 12–17 years (ELIPSE OLE) received evinacumab 15 mg/kg IV Q4W plus stable LLT. At baseline, the mean calculated LDL-C level was 301.9 mg/dL in children and 300.4 mg/dL in adolescents; most participants (95% of children and 79% of adolescents) were genetically diagnosed. Following treatment, mean baseline LDL-C levels decreased by 45% and 48% at Week 48 and 41% and 51% at Week 72 in children and adolescents, respectively. Evinacumab was also generally well tolerated [49]. TEAEs occurred in all children and 12 adolescents (86%). Treatment-related TEAEs occurred in 4 children (20%) and no adolescents.

### 3.4 Clinical trial data: safety

An analysis assessing safety data from 3 phase 3 studies of evinacumab in HoFH (ELIPSE-HoFH, ELIPSE-OLE, and Study 17,100) found that evinacumab was generally well tolerated across the 180 participants included; there were no treatment-related serious TEAEs or deaths[50]. Overall, immunogenicity was low; there were 3 reports (1.7%) of treatment-emergent antidrug antibodies (ADAs), all of which were characterized as transient with low (<1000) titers. Furthermore, there were few meaningful changes in alkaline phosphatase, alanine aminotransferase (ALT), aspartate aminotransferase (AST), and bilirubin. Four participants (2.2%) had ALT >3x upper limit of normal (ULN), 1 (0.6%) had ALT >5x ULN, and 1 (0.6%) had AST>3x ULN. In the placebo-controlled ELIPSE study, TEAEs that occurred more frequently in the evinacumab arm (*n* = 44) than in the placebo-evinacumab arm (*n* = 20) during the OLE period included influenza-like illness (4.5% vs 0), back pain (6.8% vs 0), nausea (6.8% vs 0), headache (11.4% vs 5.0%), and nasopharyngitis (11.4% vs 5.0%); there were no concerns over liver safety [24,25]. Across all studies of IV 15 mg/kg evinacumab administered to participants with HoFH or heterozygous FH (including refractory FH) and hypertriglyceridemia, treatment-related adverse events were rare; common TEAEs were mild or non-serious and included headache (9.0–16.4%), nausea (6.8–14.3%), injection-site erythema (5%), back pain (6.8–12.1%), influenza-like illness (4.5–13.8%), and nasopharyngitis (11.4–19.8%) [22,[24], [25], [26], [27], [28],^44^]. A separate analysis of infusion-related reactions (defined as adverse events occurring during or within 2 h after completion of infusion) observed in evinacumab clinical studies found that they occurred in a minority of participants and were mostly mild in severity, although there was a single report of anaphylaxis in 1 individual (see also Section 4.3.1) [51].

### 3.5 Real-world data

Limited real-world data examining evinacumab for the treatment of HoFH have been published. One single-center study in Italy examined the effect of treatment with evinacumab 15 mg/kg IV Q4W in 7 adolescent and adult participants with HoFH who were receiving concomitant lipoprotein apheresis treatment. The participants ranged in age from 14 to 63 years and 5 were female. After 24 months of treatment, all participants were transitioned into a compassionate extension period during which they received evinacumab without apheresis for approximately 12 months. After 30 and 36 months of treatment, mean LDL-C was reduced by 43.4% and 54.2%, respectively [52]. In a retrospective, multicenter study in the United States of 20 adult and 4 pediatric participants (mean age of 40 years [range 5–84 years] and mean baseline LDL-C of 309.3 mg/dL) with HoFH who received evinacumab 15 mg/kg IV Q4W, mean reductions in LDL-C of 53.2% from baseline to Week 48 were observed [45]. Across all these real-world studies, evinacumab was well-tolerated [45,52,53]. However, 1 study reported that 1 participant discontinued treatment due to a TEAE of back pain [45].

In a computer-based model, simulations were used to predict the pharmacokinetics and efficacy of evinacumab in children aged 6 months to <5 years with HoFH [54]. The model predictions provided evidence for the use of evinacumab 15 mg/kg IV Q4W in these young children. Real-world validation through a managed access program was then conducted. Six participants aged 1 to <5 years received between 5 and 23 IV infusions of evinacumab 15 mg/kg Q4W. LDL-C reductions of 41.3% to 77.3% were reported and the data were found to be consistent with the computer-based simulations, supporting the use of evinacumab in children with HoFH as young as 6 months.

Small studies have also reported regression of atherosclerotic plaques during treatment with evinacumab in combination with other LLTs [55,56]. In a study involving 9 participants with HoFH receiving intensive LLT (median age 12.4 [Q1-Q3: 10.7–17.0] years), a significant correlation between cumulative LDL-C exposure and plaque volumes (as measured by cardiac computed tomography [CT] angiography) was observed at baseline. Every 10 mmol/L (386.7 mg/dL) years of cumulative LDL-C exposure increment was associated with a 5.8% (*P* < 0.001) higher percent atheroma volume (PAV). Following initiation of evinacumab 15 mg/kg IV Q4W, it was observed that the achieved residual cumulative LDL-C exposure from baseline was significantly associated with PAV changes at 6 months. The median absolute atheroma volume at follow-up was 11.75 (Q1–Q3: 9.4–30.0) mm^3^. Furthermore, every 1 mmol/L (38.67 mg/dL) years of higher cumulative LDL-C exposure between atheroma scans was associated with a 21.4% (95% CI: 0.6–46.4%; *P* = 0.045) PAV increase from baseline. The median time-weighted LDL-C exposure after initiation of evinacumab decreased by 76.5% from 12.8 (Q1–Q3: 10.2–14.3) to 3.0 (Q1–Q3: 2.8–3.8) mmol/L per year (495.0 [394.4–553.0] to 116.0 [108.3–146.9] mg/dL per year). Participants with a time-weighted cumulative LDL-C exposure below the median (≤3.0 mmol/L [≤116.0 mg/dL] per year) showed significantly lower PAV change (−0.3 vs 1.2%, *P* = 0.03) [55].

## 4 Clinicians’ experiences with evinacumab

### 4.1 Administration

Evinacumab is administered at a dose of 15 mg/kg Q4W through IV infusion over a 60-minute period by a healthcare professional [4], either at home or in an outpatient infusion center (Table 3).

**Table 3:** Practical considerations on evinacumab for clinicians [4].

Consideration	Explanation
Mechanism of action	Evinacumab is a fully human monoclonal antibody that binds to and inhibits ANGPTL3. ANGPTL3 inhibition results in activation of LPL and EL, and subsequent reductions in LDL-C, HDL-C, and triglycerides. Evinacumab reduces LDL-C independently of the LDLR pathway, making it particularly useful for people with HoFH who have little-to-no LDLR activity
Indication	Indicated as adjunctive therapy to standard LLTs for treatment of adult and pediatric participants aged ≥1 years with HoFH in the United States and aged >6 months in other parts of the world. The safety and effectiveness of evinacumab have not been established in participants with other causes of hypercholesterolemia, including heterozygous familial hypercholesterolemia
Shipping and storage	Evinacumab 150 mg/mL is provided as 345 mg/2.3 mL or 1200 mg/8 mL solution in single-dose vials. Evinacumab should be stored in the refrigerator at 2–8 °C and protected from light. It should not be frozen or shaken. Evinacumab does not contain a preservative. If not used immediately, the diluted solution should be refrigerated at 2–8 °C for not >24 h from the time of preparation or at room temperature up to 25 °C for not >6 h from the time of infusion preparation to the end of the infusion.
Dosage and administration	Evinacumab is dosed at 15 mg/kg every 4 weeks and administered by IV infusion over 60 min by a healthcare professional. Infusion can be completed at home with home nursing or at an infusion center. The required volume of evinacumab should be withdrawn from the vial and transferred into an IV infusion bag containing a maximum volume of 250 mL of normal saline (0.9% sodium chloride injection, USP) or dextrose (5% dextrose injection, USP). The solution should be mixed by gentle inversion; do not shake. The diluted solution should be administered immediately after preparation and any unused portion left in the vial discarded.
Drug–drug interactions	No formal studies conducted. In the clinical trial setting, evinacumab did not have a meaningful effect on statin concentration in participants taking statins. As a monoclonal antibody, evinacumab is not anticipated to interact with cytochrome P450 or other drug transporters, as such, drug-drug interactions between evinacumab and other drugs are not anticipated.
Contraindications	History of serious hypersensitivity reactions, such as anaphylaxis, to evinacumab or to any of the excipients^a^ in evinacumab. Evinacumab may cause fetal harm based on animal studies. Consider pregnancy test prior to initiation of evinacumab. Advise women of childbearing potential to use contraception during treatment and for at least 5 months following the last dose.
Common AEs	Nasopharyngitis, influenza-like illness, dizziness, rhinorrhea, nausea, and fatigue,

a L-arginine hydrochloride, l-histidine, l-histidine monohydrochloride monohydrate, l-proline, polysorbate 80, and water for injection, USP. AE, adverse event; ANGPTL3, angiopoietin-like protein 3; EL, endothelial lipase; HDL-C, high-density lipoprotein cholesterol; HoFH, homozygous familial hypercholesterolemia; IV, intravenous; LDL-C, low-density lipoprotein cholesterol; LDLR, low-density lipoprotein receptor; LLT, lipid-lowering therapy; LPL, lipoprotein lipase.

**Box 1:** Considerations for HoFH diagnosis.

HoFH diagnoses can be made using either **clinical** or **genetic** criteria [9,12]. • **Clinical**: Presence of LDL-C > 400 mg/dL, appearance of xanthomas in pediatric age (typically before age 10 years), and/or LDL-C levels compatible with HeFH in both parents, together with the absence of secondary causes • **Genetic**: Presence of 2 pathogenic variants in separate alleles of *LDLR* (85–90% of cases), *APOB* (5–10%), *PCSK9* (1–3%), or biallelic pathogenic variants in *LDLRAP1* (<1%) **The threshold for making a clinical diagnosis of HoFH typically requires an untreated LDL-C level ≥400mg/dL.** However, clinical criteria for the diagnosis of HoFH are limited in key respects, such as the unavailability or unreliability of family history of familial hypercholesterolemia, premature ASCVD, or the presence of xanthomas. Each set of criteria also places different emphases on LDL-C thresholds, family history, genetic testing, and physical signs of HoFH, which can lead to inconsistent identification of people with likely HoFH. Additionally, it may be difficult to ascertain untreated LDL-C levels in individuals who began LLT at a young age. A recent publication by Gu et al. (2025) described a simulation model to estimate the untreated LDL-C value of people receiving a standard regimen of LLTs, which may aid diagnosis. For example, for a person receiving a statin, an ezetimibe, and a PCSK9 inhibitor, with a treated LDL-C level of 250 mg/dL, the calculator suggests an untreated value of 542.5 mg/dL (95% confidence interval, 438.6–735.3), which is suggestive of HoFH [57].**Genetic diagnosis of HoFH is made by establishing a pathogenic variant in the *LDLR, LDLRAP1, APOB*, or *PCSK9* genes** [9,12]. However, availability of genetic testing may be limited particularly in low-income countries. Negative genetic testing should not rule out HoFH (especially as not all causal genetic variants are known) in individuals who meet clinical criteria for HoFH; a clinical diagnosis of HoFH does not require genetic confirmation.Recognizing which individuals with suspected HoFH are candidates for evinacumab treatment may facilitate the introduction of adequate levels of therapy once a diagnosis is made.

ASCVD, atherosclerotic cardiovascular disease; HoFH, homozygous familial hypercholesterolemia; LDL-C, low-density lipoprotein cholesterol; LDLR, low-density lipoprotein receptor; LLT, lipid-lowering therapy; PCSK9, proprotein convertase subtilisin/kexin type 9.

Evinacumab is shipped in single vials containing either 345 mg/2.3 mL or 1200 mg/8 mL, with each mL containing 150 mg of evinacumab for dilution and administration. The vials should be stored in a refrigerator at 2–8 °C and protected from light [4]. The required volume of evinacumab should be extracted from the vial and transferred into an IV infusion bag of 0.9% sodium chloride injection or 5% dextrose injection. The final concentration of the diluted solution should be between 0.5 mg/mL and 20 mg/mL depending on the individual’s current body weight: in those weighing 3 kg to <26 kg the maximum diluent volume should be 5 mL/kg; in those weighing 26 kg to <45 kg the maximum diluent volume should be 150 mL; and in those weighing ≥45 kg the maximum diluent volume should be 250 mL [4].

### 4.2 Concomitant treatment with evinacumab

While individuals with HoFH are likely to have received a variety of pharmaceutical interventions prior to initiating evinacumab, care should be taken to counsel people beginning treatment, as some may not have previously received treatment with infused monoclonal antibodies. Individuals should continue treatment with standard LLTs during evinacumab treatment. LDL-C lowering goals should be targeted based on current guidelines. The current NLA, EAS, and ACC/AHA joint guidelines recommend the following LDL-C levels based on ASCVD status: <70 mg/dL for those without ASCVD, <55 mg/dL for those with ASCVD, and <40 mg/dL for those with ASCVD and recurrent coronary symptoms, with the overall goal of lowering LDL-C as much as possible to mitigate the lifetime risk of ASCVD caused by the cumulative exposure to elevated LDL-C levels [9,12,15,34].

Although no formal drug–drug interaction studies have been carried out, in the clinical trial setting, the concentrations of statins were not meaningfully altered prior to and post evinacumab treatment [4]. As a monoclonal antibody, evinacumab is not anticipated to interact with cytochrome P450 or other drug transporters, as such, drug-drug interactions between evinacumab and other drugs are not anticipated. Clinical data and case reports have also examined the use of evinacumab in conjunction with other LDLR-independent therapies, such as lipoprotein apheresis and lomitapide, with no evident safety concerns [37,58,59]. In the phase 3 ELIPSE study, most participants were receiving evinacumab alongside a statin (94%); others were receiving evinacumab plus a PCSK9 inhibitor (77%), ezetimibe (75%), or lomitapide (25%) or were also undergoing apheresis (34%) [24]. The percent changes in LDL-C levels from baseline to Week 24 were consistent across the range of background therapies received. Several other case reports have also examined the use of evinacumab in combination with LLT regimens [58,59].

Evinacumab can be administered after apheresis during the same clinic visit [4,60]; administration before could potentially result in a reduction in evinacumab plasma concentration. In a pooled subanalysis of data from 138 participants with a mean (SD) age of 34.5 (18.0) years across the entire evinacumab HoFH clinical development program investigating apheresis frequency by baseline lipoprotein apheresis status, it was found that at least 29% of participants who were receiving lipoprotein apheresis at study baseline were able to decrease lipoprotein apheresis frequency during the study. Administration of evinacumab and lipoprotein apheresis during the same clinic visit may allow for a reduction in the lipoprotein apheresis burden [37], which may be desirable to some individuals for whom frequent lipoprotein apheresis presents a considerable burden, such as those who live a far distance from their nearest treatment center. In a single-center case series in Austria 4 of 5 participants aged 10–19 years with HoFH who received evinacumab 15 mg/kg IV Q4W concomitantly with apheresis experienced large enough reductions in LDL-C to consider reducing apheresis frequency; 1 participant was able to reduce apheresis frequency from weekly to three times per month [53]. These results are also supported by published case studies. In one such case study, a 17-year-old male with HoFH had previously received a regimen of statin, ezetimibe, PCSK9 inhibitor, lomitapide, and lipoprotein apheresis, with limited reduction in LDL-C. Evinacumab was added to the regimen and he was able to reduce the frequency of lipoprotein apheresis [58]. Another case study examined a 47-year-old female with HoFH who initiated a regimen of therapeutic plasma exchange (following an anaphylactoid-like reaction during a prior apheresis session). After adding evinacumab to her treatment regimen, she was able to decrease the frequency of therapeutic plasma exchange from twice a month to once a month while maintaining LDL-C reduction [59].

### 4.3 Management of individuals during evinacumab treatment

#### 4.3.1 Infusion-related reactions

Infusion-related reactions occur in ∼7% of people treated with evinacumab and include infusion-site pruritus, pyrexia, muscular weakness, nausea, and nasal congestion. In the authors’ experience, pre-medication with IV administration of 25 mg diphenhydramine to prevent reactions in susceptible individuals has proven useful. The prescribing information recommends infusing evinacumab over a 60-minute period, however, slowing the rate of infusion can also mitigate infusion-related symptoms and should be considered, if appropriate [51,61,62]. A very small minority of individuals may experience serious hypersensitivity reactions with evinacumab treatment including anaphylaxis, which can be treated according to the standard of care; including epinephrine in severe cases, and discontinuation of therapy [4]. Pretreatment with prednisone or antihistamines (diphenhydramine and/or famotidine) may be considered to minimize the risk of hypersensitivity reactions. In a review of infusion-related reactions following evinacumab treatment in over 380 adults and children with HoFH, there was a single report of anaphylaxis in 1 individual [51].The individual recovered with oral antihistamine and discontinued treatment.

#### 4.3.2 Pregnancy and lactation

Data from animal reproduction studies suggest that evinacumab may cause fetal harm; therefore, females of reproductive age should not use evinacumab during pregnancy and should use an effective method of birth control during treatment and for at least 5 months after their last dose [4]. A pregnancy test for females of reproductive age at time of initiation of therapy is also recommended. There are no data on the presence of evinacumab in human breastmilk, the effects on the breastfed infant, or the effects on milk production. In an analysis of safety across 3 phase 3 studies of evinacumab in HoFH (ELIPSE-HoFH, ELIPSE-OLE, and Study 17,100), 5 pregnancies with exposure in the first trimester were reported; 4 were in female participants and 1 was in the partner of a male participant[50]. All 4 females discontinued evinacumab treatment. One participant was determined to be approximately 1 month pregnant shortly after starting open-label treatment in ELIPSE-HoFH. She had been randomized to evinacumab on study day 1 and was taking hormonal contraception; she stopped study treatment but remained in the study, completing all study visits. The pregnancy was completed to term with no complications and the baby was born via a vaginal delivery in good general health. The other 3 females were participants in the ELIPSE-OLE study; 2 permanently discontinued evinacumab for the entire duration of the study and 1 stopped evinacumab after the positive pregnancy test but reinitiated treatment giving birth. All pregnancy outcomes were normal with no embryofetal toxicities observed.

#### 4.3.3 Pediatrics

Many people with HoFH are identified during childhood [12]; treatment should be initiated at time of diagnosis to prevent premature ASCVD and the high rate of mortality associated with HoFH if left untreated or insufficiently treated. The 2026 NLA consensus recommends LDL-C goals of <100 mg/dL for primary prevention and <70 mg/dL in those with evidence of ASCVD in children and adolescents with HoFH [9]; the 2023 EAS guideline recommends an LDL-C goal of <115 mg/dL in those without ASCVD and a lower goal in those with ASCVD [12]; and the 2026 ACC/AHA guideline recommends an LDL-C of <130 mg/dL in primary prevention and <100 mg/dL in those with additional risk factors [15]. LLT is generally well-tolerated in children and adolescents with FH [9], and current guidelines recommend novel therapies (e.g. evolocumab), including LDLR-independent treatment (e.g. evinacumab) for children and adolescents with HoFH [9,12]. They also note consideration of liver transplant if lipoprotein apheresis or novel therapies are not available [9,12]. Evinacumab is authorized for use in children as young as 6 months old by the European Medicines Agency and 1 year old by the US Food and Drug Administration [4]. The safety profile of evinacumab in children and adolescents is similar to that in adults [3].

### 4.4 General considerations when managing people with HoFH

Cascade screening of all first- and second-degree relatives is essential for all people diagnosed with HoFH and is recommended in current guidance from NLA, EAS, and the ACC/AHA [9,12,15]. Identification of individuals with HoFH through cascade screening is the most cost-effective strategy for identifying new cases of both HoFH and heterozygous FH and facilitates earlier diagnosis and treatment, thereby reducing the risk of ASCVD and early mortality [9].

Given that people with HoFH have high exposure to atherogenic lipoproteins from birth and subsequently a high lifetime risk of atherosclerosis, current guidelines from EAS and NLA strongly recommend that detailed cardiovascular imaging of the heart and aorta is conducted at baseline [9,12]. Children and adults with HoFH frequently develop aortic valve stenosis and severe coronary artery stenosis that may be asymptomatic until an event or sudden death [9,12]. People with HoFH should receive echocardiographic evaluation of the heart and aorta at baseline and annually thereafter [12]. CT angiography should be performed at least once in individuals 3 years of age and older and as clinically indicated thereafter, taking into consideration baseline disease, LDL-C lowering, radiation risk, and cost [12]. Invasive coronary angiography is indicated in individuals with symptoms and/or signs suggestive of ischemia or valve malfunction [12]. CT angiography with fractional flow and plaque analysis (particularly for those without a history of stenting or coronary bypass) or vascular studies may assist in establishing further cardiovascular treatment and lipid goal management needs.

### 4.5 Limitations and future perspectives

There remain limited real-world studies examining the long-term benefit of evinacumab in individuals with HoFH. Due to the limited number of people with confirmed HoFH globally, it is difficult to establish a comprehensive real-world evidence base for the use of evinacumab in all clinically relevant scenarios. Further high-quality real-world evidence is required to confirm the long-term efficacy and safety of evinacumab. HoFH remains underdiagnosed globally, and improved efforts for diagnoses are also needed to identify those who may be likely to benefit from HoFH-specific therapies like evinacumab. Unfortunately, even a diagnosis does not guarantee access to evinacumab or similar treatments, which may be limited by high cost, reimbursement challenges, geographic disparities, and availability primarily through specialized treatment centers in some countries [[63], [64], [65], [66]]. The results observed for evinacumab in both clinical trial and real-world settings support the continued investigation of ANGPTL3-targeted therapies, including future ANGPTL3 gene-editing approaches, for the management of all forms of dyslipidemia.

## 5 Conclusions

Clinical trial and real-world evidence demonstrate clear benefit for the use of evinacumab in the treatment of children, adolescents, and adults with HoFH who are unable to meet their LDL-C treatment goals with standard LLTs alone. Evinacumab inhibits ANGPTL3 and lowers LDL-C by approximately 50% including in individuals with HoFH who have an absence of functional LDLR activity. Further, while limited in number, recent case reports show that evinacumab can be used in conjunction with other LLTs, including lipoprotein apheresis and lomitapide, without compromising the safety or efficacy of either treatment. Research has shown that HoFH should be treated early and aggressively to minimize the risk of ASCVD caused by lifelong elevated LDL-C levels. A holistic management approach should be adopted, which takes into account periods of treatment disruption/interruption, such as during pregnancy. More recently, it has been shown that evinacumab treatment may also reverse some atherosclerosis, as evidenced by case reports of plaque and atheroma regression. Taken together, the available data support the role of evinacumab as a versatile and potent therapeutic option for reducing LDL-C in children and adults with HoFH.

## 6 Discussion

**Figure:**
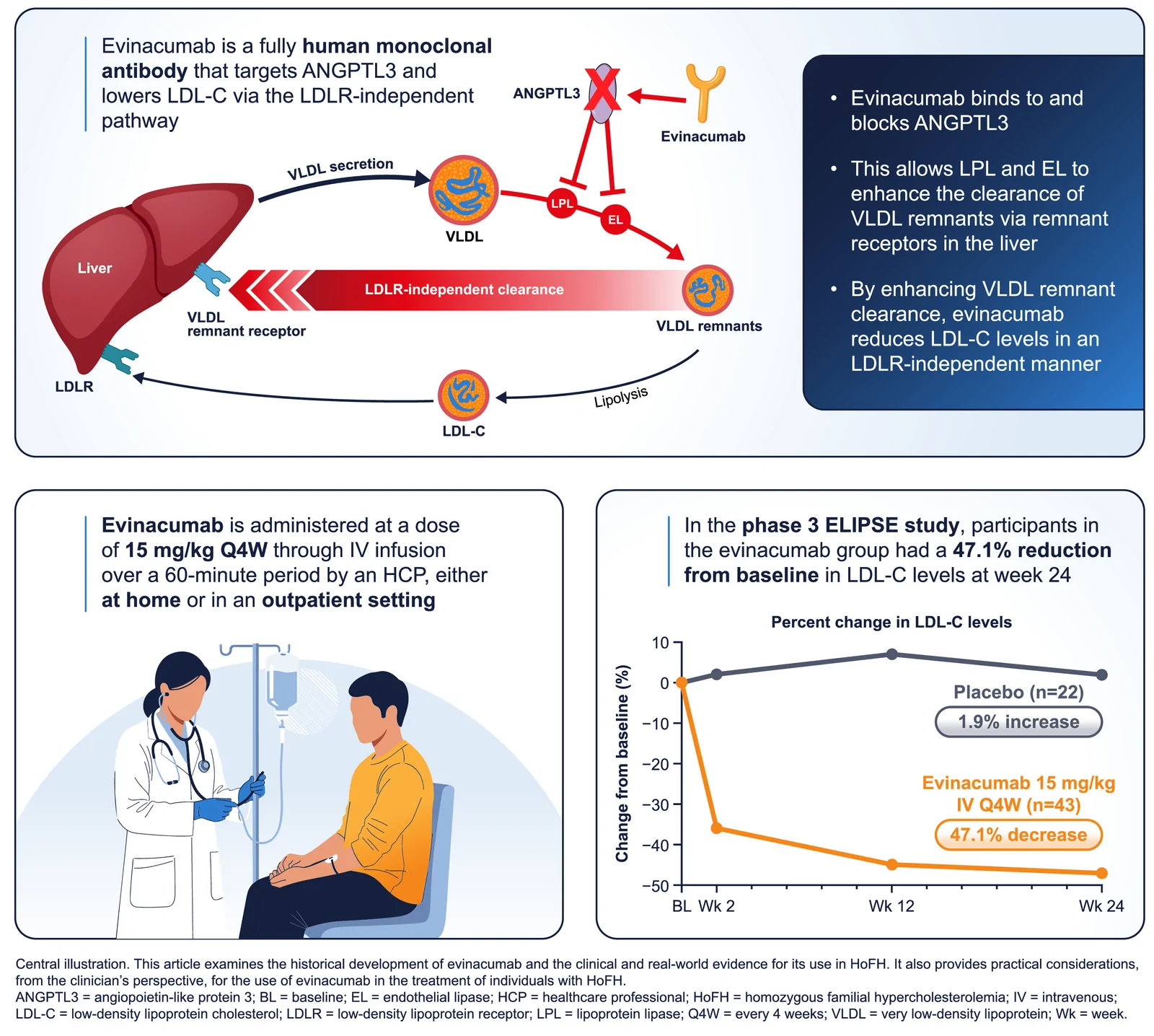
Central illustration.

## Funding

This study was funded by Regeneron Pharmaceuticals, Inc. Regeneron Pharmaceuticals, Inc., was involved in the study design, writing, and decision to submit this article for publication.

## CRediT authorship contribution statement

**Archna Bajaj:** Writing – review & editing, Supervision, Methodology, Conceptualization. **Michael McGuire:** Writing – review & editing, Writing – original draft, Project administration, Methodology, Conceptualization. **Suzanne Shugg:** Writing – review & editing, Methodology. **Richard Godin:** Writing – review & editing, Writing – original draft, Project administration, Methodology, Funding acquisition, Conceptualization. **James A. Underberg:** Writing – review & editing, Methodology, Conceptualization.

## Declaration of competing interest

The authors declare the following financial interests/personal relationships which may be considered as potential competing interests: Archna Bajaj reports financial support was provided by Regeneron Pharmaceuticals, Inc. Michael McGuire reports financial support was provided by Regeneron Pharmaceuticals, Inc. Suzanne Shugg reports was provided by Regeneron Pharmaceuticals, Inc. Richard Godin reports financial support was provided by Regeneron Pharmaceuticals, Inc. James A. Underberg reports financial support was provided by Regeneron Pharmaceuticals, Inc. Archna Bajaj reports a relationship with Alexion that includes: funding grants. Archna Bajaj reports a relationship with Amgen that includes: funding grants. Archna Bajaj reports a relationship with Arrowhead Pharmaceuticals that includes: consulting or advisory and funding grants. Archna Bajaj reports a relationship with Eli Lilly that includes: funding grants. Archna Bajaj reports a relationship with Ionis that includes: consulting or advisory and funding grants. Archna Bajaj reports a relationship with NewAmsterdam Pharma that includes: funding grants. NewAmsterdam Pharma reports a relationship with Novartis that includes: funding grants. Archna Bajaj reports a relationship with Chiesi that includes: consulting or advisory. Michael McGuire reports a relationship with Regeneron Pharmaceuticals, Inc. that includes: employment and equity or stocks. Richard Godin reports a relationship with Regeneron Pharmaceuticals, Inc. that includes: employment and equity or stocks. James A. Underberg reports a relationship with Amgen that includes: consulting or advisory and speaking and lecture fees. James A. Underberg reports a relationship with Arrowhead that includes: speaking and lecture fees. James A. Underberg reports a relationship with Esperion that includes: speaking and lecture fees. James A. Underberg reports a relationship with Ionis that includes: speaking and lecture fees. James A. Underberg reports a relationship with Regeneron Pharmaceuticals, Inc. that includes: speaking and lecture fees. James A. Underberg has contracted research from Chiesi; is a steering committee member for Aegerion LOWER Registry Trial Data Monitoring Committee; and is on the Clinical Data Monitoring Committee for Marea Pharma. If there are other authors, they declare that they have no known competing financial interests or personal relationships that could have appeared to influence the work reported in this paper.
