# Supplementary material for: Clinical considerations on the use of evinacumab for the treatment of homozygous familial hypercholesterolemia
Source: Am J Prev Cardiol. 2026 Aug 11;30:101790. doi: 10.1016/j.ajpc.2026.101790 (PMC13632010; doi:10.1016/j.ajpc.2026.101790)
Supplement: Supplementary file 1 [file mmc1.pdf]

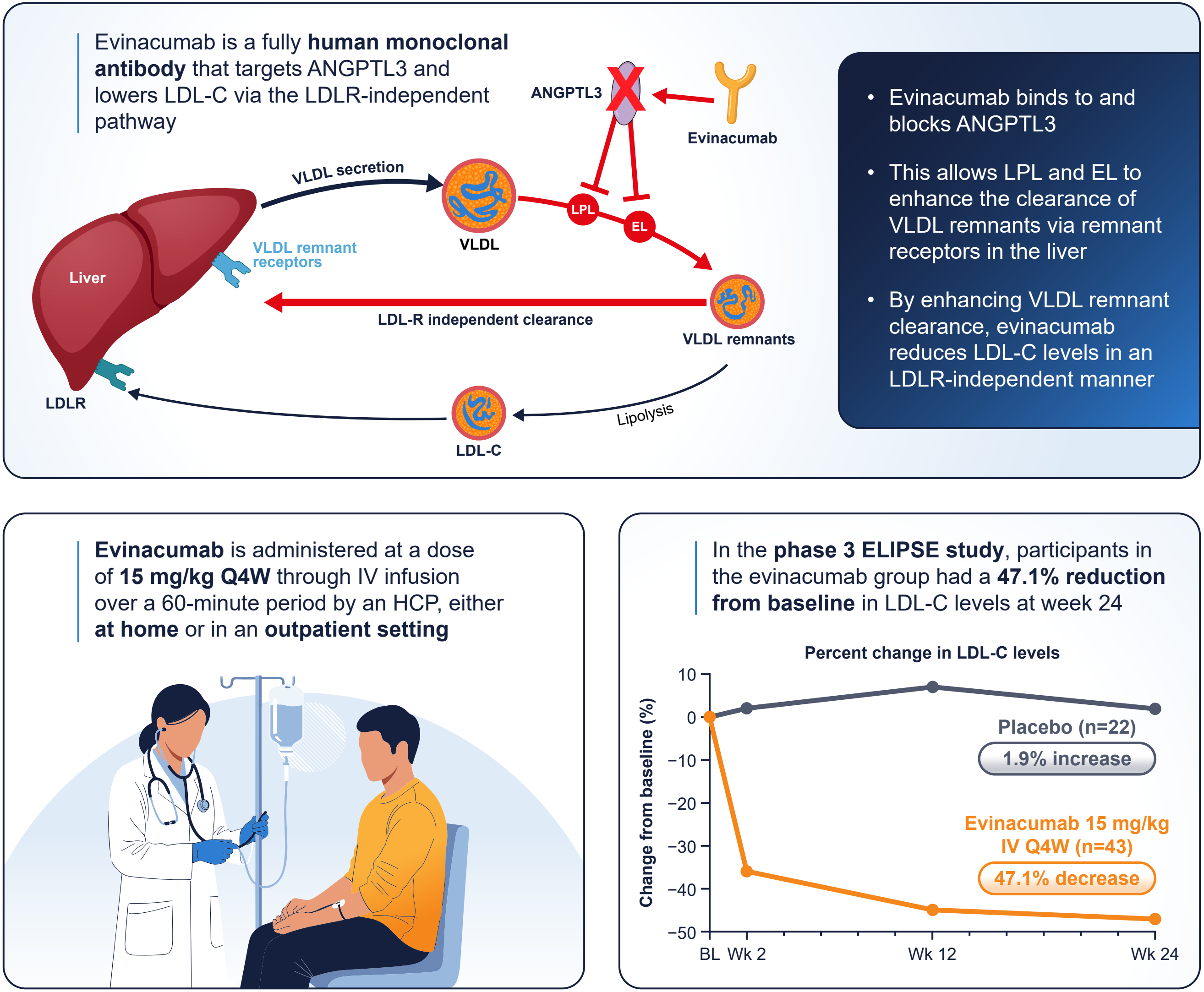


Central illustration. This article examines the historical development of evinacumab and the clinical and real-world evidence for its use in HoFH. It also provides practical considerations, from the clinician's perspective, for the use of evinacumab in the treatment of individuals with HoFH.

ANGPTL3 = angiopoietin-like protein 3; BL = baseline; EL = endothelial lipase; HCP = healthcare professional; HoFH = homozygous familial hypercholesterolemia; IV = intravenous; LDL-C = low-density lipoprotein cholesterol; LDLR = low-density lipoprotein receptor; LPL = lipoprotein lipase; Q4W = every 4 weeks; VLDL = very low-density lipoprotein; Wk = week.
